# Aggregation-Induced Emissive Scintillators: A New Frontier for Radiation Detection and Imaging

**DOI:** 10.1007/s40820-025-01671-x

**Published:** 2025-02-24

**Authors:** Xinyi Li, Jiafu Yu, Yinghao Fan, Yuting Gao, Guangda Niu

**Affiliations:** 1 Engineering Research Center of Nano-Geomaterials of Ministry of Education, Faculty of Material Science and Chemistry, China University of Geosciences, Wuhan, 430074 People’s Republic of China; 2 State Key Laboratory of Biocatalysis and Enzyme Engineering, School of Life Sciences, Hubei University, Wuhan, 430062 People’s Republic of China; 3 Wuhan National Laboratory for Optoelectronics and School of Optical and Electronic Information, Huazhong University of Science and Technology, Wuhan, 430074 People’s Republic of China

**Keywords:** Aggregation-induced emission, Scintillators, Radiation detection, Radiation imaging

## Abstract

Aggregation-induced emission (AIE) is a unique phenomenon where certain organic materials exhibit enhanced luminescence in their aggregated states, overcoming the typical quenching observed in conventional organic materials. Since its discovery in 2001, AIE has driven significant advances in fields like OLEDs and biological imaging, earning recognition in fundamental research. However, its application in high-energy radiation detection remains underexplored. Organic scintillators, though widely used, face challenges such as low light yield and poor radiation attenuation. AIE materials offer promising solutions by improving light yield, response speed, and radiation attenuation. This review summarizes the design strategies behind AIE scintillators and their very recent applications in X-ray, γ-ray, and fast neutron detection. We highlight their advantages in enhancing detection sensitivity, reducing background noise, and achieving high-resolution imaging. By addressing the current challenges, we believe AIE materials will play a pivotal role in advancing future radiation detection and imaging technologies.

## Highlights

- Summarizes the high quantum efficiency and rapid response of aggregation-induced emission (AIE) materials in radiation detection, showcasing their advantages in high-energy radiation signal detection.
- Reviews the progress in AIE materials’ radiation response for efficient detection of X-rays, γ rays, neutrons, and other radiation types.
- Long term stability, device integration, and adaptability to diverse radiation forms remain key challenges for broader AIE material applications.

## Graphical abstract

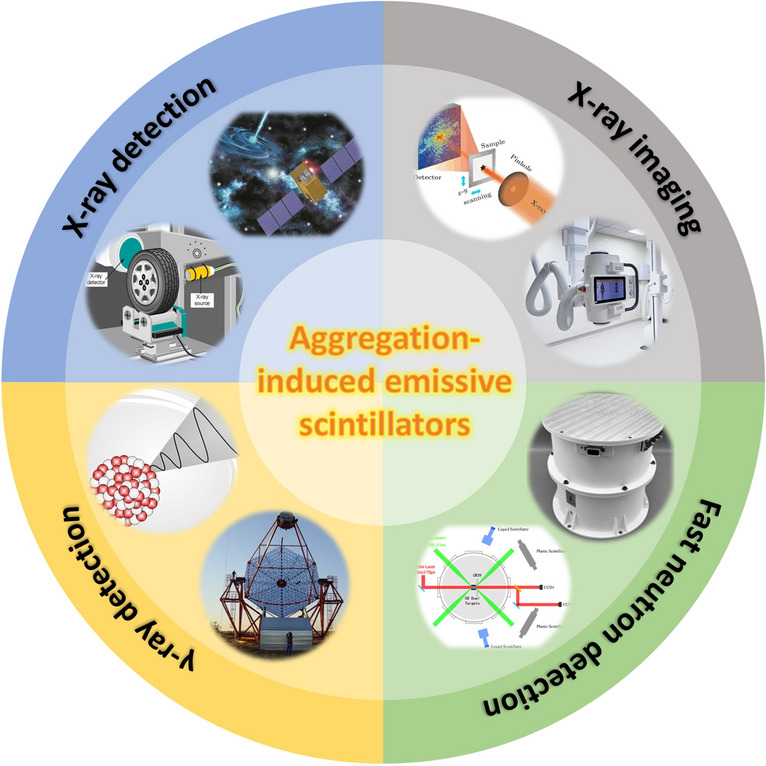

## Introduction

Radiation detection and imaging technologies are widely utilized in fields such as medical diagnostics [1], high-energy physics, non-destructive testing [2, 3], and security surveillance [4]. Central to these technologies are radiation detectors, which convert radiation into measurable signals [5]. Scintillator-based radiation detection is the most widely used approach today, owing to the fast response speed, ease of fabrication, integration capability, and low cost of scintillators [6]. Scintillation is the process by which ionizing radiation interacts with scintillator materials to produce photons [7]. The ongoing goal in this field is to develop scintillators with higher efficiency and faster timing properties. Higher efficiency allows for the generation of more photons from a given radiation dose, improving image quality without increasing patient exposure. Fast scintillation not only enables quicker imaging with less lag, but also plays a crucial role in applications such as computed tomography (CT), positron emission tomography (PET), and high-energy physics experiments [8–10].

The development of radiation detection scintillators dates back to the late nineteenth century, with early advancements such as calcium tungstate (CaWO_4_) and zinc sulfide (ZnS)-based powders that efficiently convert X-rays into visible light [11, 12]. Since then, various inorganic single crystals and powders have been developed, many of which exhibit outstanding performance and are now widely used. While inorganic scintillators are known for their high light yield and effectiveness in detecting high-energy radiation, they still face several challenges. For example, common scintillators like NaI and CsI are prone to instability due to their hygroscopic nature [13, 14]. Achieving both high sensitivity and temporal resolution is also difficult. Scintillators like BaF_2_ offer high temporal resolution but suffer from low exciton utilization, reducing X-ray sensitivity [15]. Meanwhile, rare-earth-based scintillators like SrI_2_ have high exciton utilization but slow d → f transitions, limiting their temporal resolution [16]. Additionally, doped ion-based scintillators, such as CsI, often experience lattice defects, leading to prolonged afterglow and reduced temporal resolution [17]. Overcoming these challenges is essential to enhancing scintillator performance for radiation detection.

In recent years, organic scintillators have gained significant attention due to their advantages, including low-cost raw materials, ease of modification and processing, and the potential for large-area fabrication, making them highly promising for applications in radiation detection and imaging. However, traditional planar-structured organic scintillators often suffer from reduced emission intensity caused by π-π stacking interactions in the solid and aggregated states, which negatively impact their radiation detection performance [18]. The discovery of the aggregation-induced emission (AIE) phenomenon by Tang and colleagues has fundamentally changed the fundamental understanding of photoluminescence in organic compounds [19]. Unlike the aggregation-caused quenching (ACQ) effect, which leads to a decrease in emission intensity when many luminescent materials aggregate, AIE materials exhibit enhanced light emission in the aggregated state. This breakthrough has opened up exciting possibilities for research in organic electronics, including light-emitting diodes [20–22], sensors [23, 24], and other advanced technologies. However, despite its substantial progress, the application of AIE materials in high-energy radiation detection remains a largely untapped area.

**Fig. 1:**
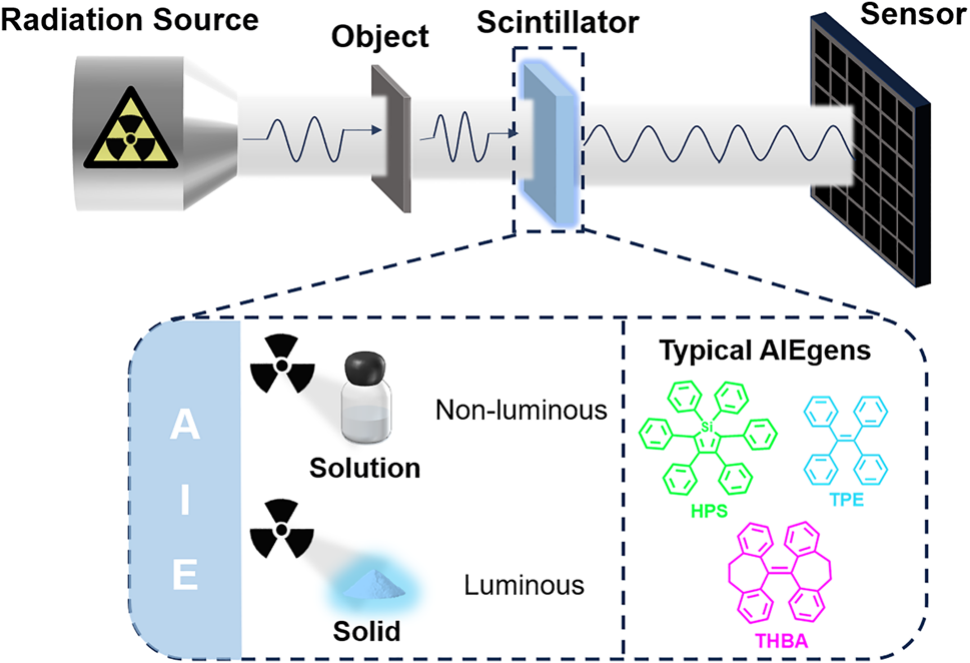
Aggregation-induced luminescent scintillators for radiation detection and imaging applications

Very recently, a range of AIE-based scintillators has been studied for potential application of radiation detection and imaging. These scintillators offer several advantages, including enhanced detection sensitivity, reduced background noise, and high-resolution imaging. This review will explore the working principles and design strategies of AIE materials, examining their applications in the detection of X-rays, γ-rays, and fast neutrons, as well as in radiation imaging technologies. Additionally, the review will address the opportunities and challenges associated with AIE scintillators in radiation detection and imaging, highlighting potential directions for future development (Fig. 1).

## Design Strategy of Aggregation-Induced Emission Scintillators

**Fig. 2:**
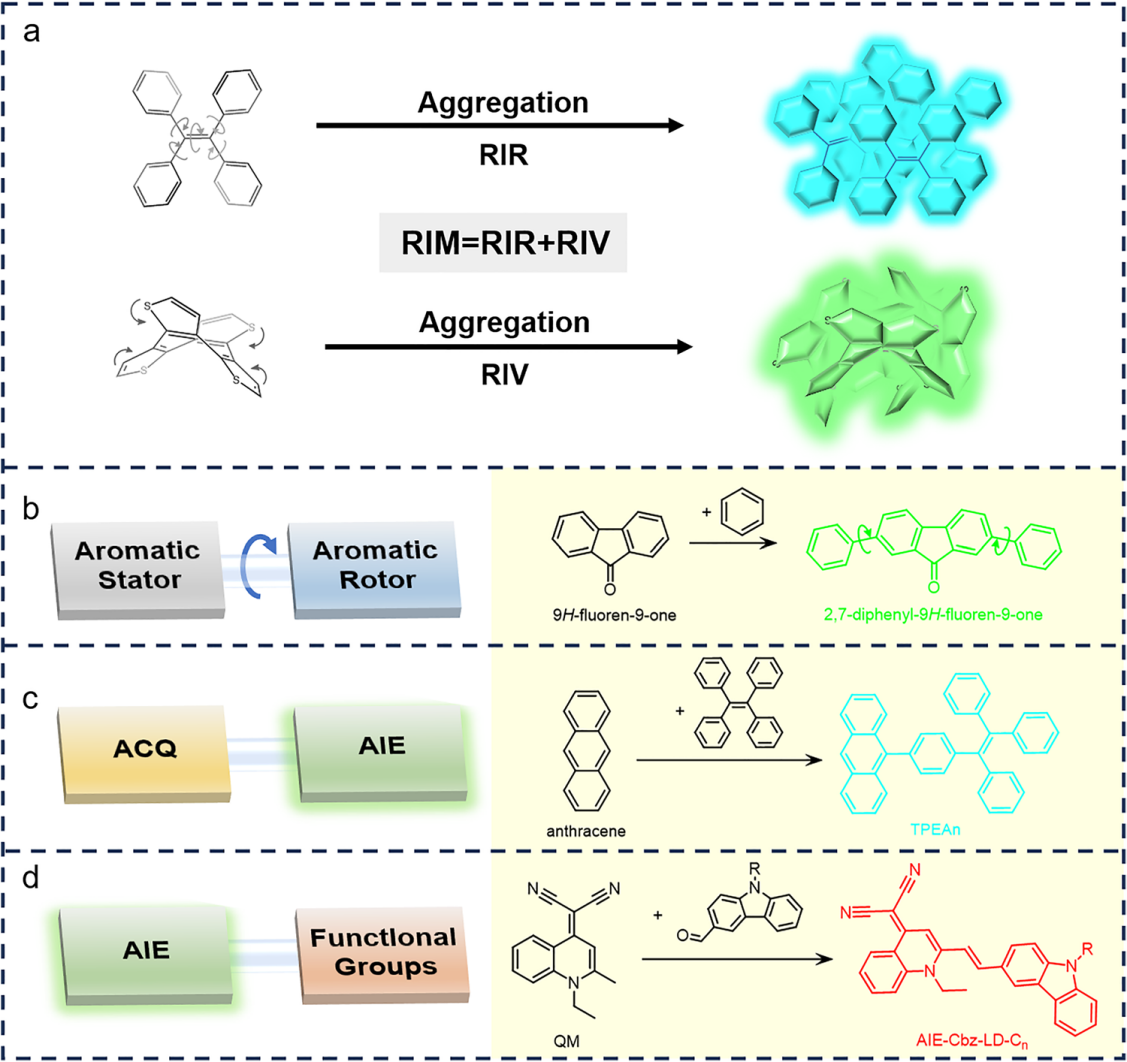
**a** As shown above, tetraphenylethylene (TPE) is non-emissive when dissolved, but becomes emissive on polymerisation due to the restriction of intramolecular rotation (RIR). In the figure below, cyclooctyl tetrathiophene (COTh) shows AIE activity in the aggregated state due to restriction by intramolecular vibration (RIV). **b**–**d** Schematic diagram for constructing an AIE molecule

As shown in Fig. 2a, the core mechanism underlying AIE is the restriction of intramolecular motion (RIM) [25, 26]. A key component of the RIM mechanism is the restriction of intramolecular rotation (RIR), which notably affects flexible units such as benzene rings and alkenes. In solution, the free rotation of these units results in energy dissipation, while in the aggregated state, steric hindrance or intermolecular stacking restricts rotational motion, thereby minimizing non-radiative decay. Similarly, the restriction of intramolecular vibration (RIV) reduces non-radiative relaxation in the aggregated state by limiting the vibrational degrees of freedom. Furthermore, AIE-active molecules are specifically designed to avoid detrimental π-π stacking interactions, which are commonly associated with fluorescence quenching in aggregated systems. Instead, these molecules preferentially engage in weaker interactions, such as C–H…π, even in the aggregated state, effectively preventing the fluorescence quenching typically observed in ACQ processes. Collectively, these factors contribute to the enhanced luminescent efficiency of AIE molecules in their aggregated form.

Building on this core mechanism, the design of AIE molecules requires careful consideration of molecular structures to optimize the RIM effect. As illustrated in Fig. 2b, the process begins with the selection of core fluorophores that exhibit strong fluorescence in their monomeric state. These fluorophores typically consist of conjugated π-electron systems, such as benzene, naphthalene, anthracene, or BODIPY, which are capable of absorbing light and re-emitting it as fluorescence [27]. However, in solution, these fluorophores often demonstrate weak or negligible fluorescence due to non-radiative decay processes, such as the rotational or vibrational motions of flexible molecular segments.

To overcome this fluorescence quenching in solution, the design of AIE molecules typically involves the incorporation of rigid or bulky substituents, such as aromatic rings, alkyl chains, or extended aromatic fragments. These substituents play a crucial role in restricting the internal flexibility of the molecules. Specifically, rotatable single bonds linking rigid aromatic “stators” with flexible “rotors” introduce a non-planar structure. The twisted geometry of the rotors prevents the close π-π stacking interactions between adjacent luminophores, thereby avoiding fluorescence quenching in the aggregated state. This structural modification effectively suppresses non-radiative decay processes in the aggregated form, as the restricted motion of the molecular segments prevents excited-state energy from dissipating through rotational or vibrational motions, thus enabling more efficient radiative decay [28]. A notable example of this strategy is the work by Wang’s research group, where two phenyl rings were attached to fluorenone to create an AIE luminophore (Fig. 2b) [29]. This luminophore shows negligible emission in solution but exhibits bright green fluorescence in the crystalline state.

Similarly, the chemical modification of ACQ scaffolds by incorporating AIE-active groups can lead to the development of new AIE materials (Fig. 2c). For instance, Tang’s research group introduced freely rotating tetraphenylethylene (TPE) into the traditional ACQ molecule anthracene [30]. This modification restricts intramolecular rotation, resulting in the molecule TPEAn exhibiting aggregation-induced emission (AIE) behavior in the condensed phase. Furthermore, chemical modifications of AIE molecules, such as the incorporation of functional groups, the integration of AIE units into polymer backbones or side chains, or the embedding of AIE-active molecules into host–guest systems, offer alternative strategies for the design of novel AIE materials (Fig. 2d) [31]. A notable example is the work by Zhu and colleagues, who synthesized a series of AIE fluorescent probes, AIE-Cbz-LD-C_n_, by conjugating quinoline with carbazole as the core unit [32]. The inclusion of the QM group in AIE-Cbz-LD-C_n_ imparts strong AIE activity, highlighting the versatility of these modifications. These strategies significantly enhance the applicability of AIE compounds, enabling their use in a wide range of applications, including sensors, optoelectronic devices, and imaging technologies.

The unique luminescent properties of AIE materials enable their potential use in radiation detection and imaging technologies. For effective detection of X-rays and γ-rays, materials must interact efficiently with these high-energy photons through either absorption or scattering, and subsequently convert the absorbed energy into a detectable signal. Therefore, the design of AIE molecules with conjugated π-systems is essential for enhancing the absorption of high-energy X-ray and γ photons. Conjugation promotes electronic delocalization within the molecule, which improves charge transport and enhances the material’s ability to interact with ionizing radiation, thereby facilitating energy transfer and boosting luminescent efficiency [33]. Furthermore, heavy atoms with high atomic numbers are known to possess stronger photon absorption and scattering capabilities [34, 35]. Thus, incorporating heavy atoms such as halogens or metals into scintillation materials can further enhance their ability to absorb X-ray photons, improving the overall detection performance of the scintillators.

The detection mechanism for fast neutrons differs significantly from that of X-rays and γ-rays. Unlike these high-energy photons, fast neutrons do not interact directly with electrons to produce electromagnetic radiation [36]. Instead, they primarily interact with matter through nuclear reactions. To detect fast neutrons, organic materials must efficiently convert the neutrons’ kinetic energy into secondary particles or radiation. Therefore, the incorporation of hydrogen-rich units in AIE molecules is essential for enhancing neutron detection. Since hydrogen atoms have a mass similar to that of neutrons, they are particularly effective in elastic scattering events. This enables the transfer of the neutron’s kinetic energy to hydrogen atoms, which then emit secondary radiation, facilitating detection.

Through these strategies, the design of AIE materials can be optimized for different types of radiation detection, enhancing X-ray and γ-ray detection efficiency through improved photon absorption, or boosting fast neutron detection efficiency by incorporating hydrogen-rich structures. This targeted design approach enables AIE materials to possess broad application potential in the fields of radiation detection and imaging.

## Application of AIE Scintillators in X-Ray Detection and Imaging

### X-Ray Detection

AIE materials have garnered significant attention as scintillators for X-ray detection, primarily due to their efficient conversion of X-ray energy into visible light. These materials offer advantages such as high light yields, low detection limits, and a competitive edge over traditional scintillators. Recent advancements further demonstrate their potential to enhance X-ray detection capabilities and drive progress in radiation imaging technologies. In the following section, we highlight key developments in AIE-based scintillators, emphasizing their innovative contributions to the field.

**Fig. 3:**
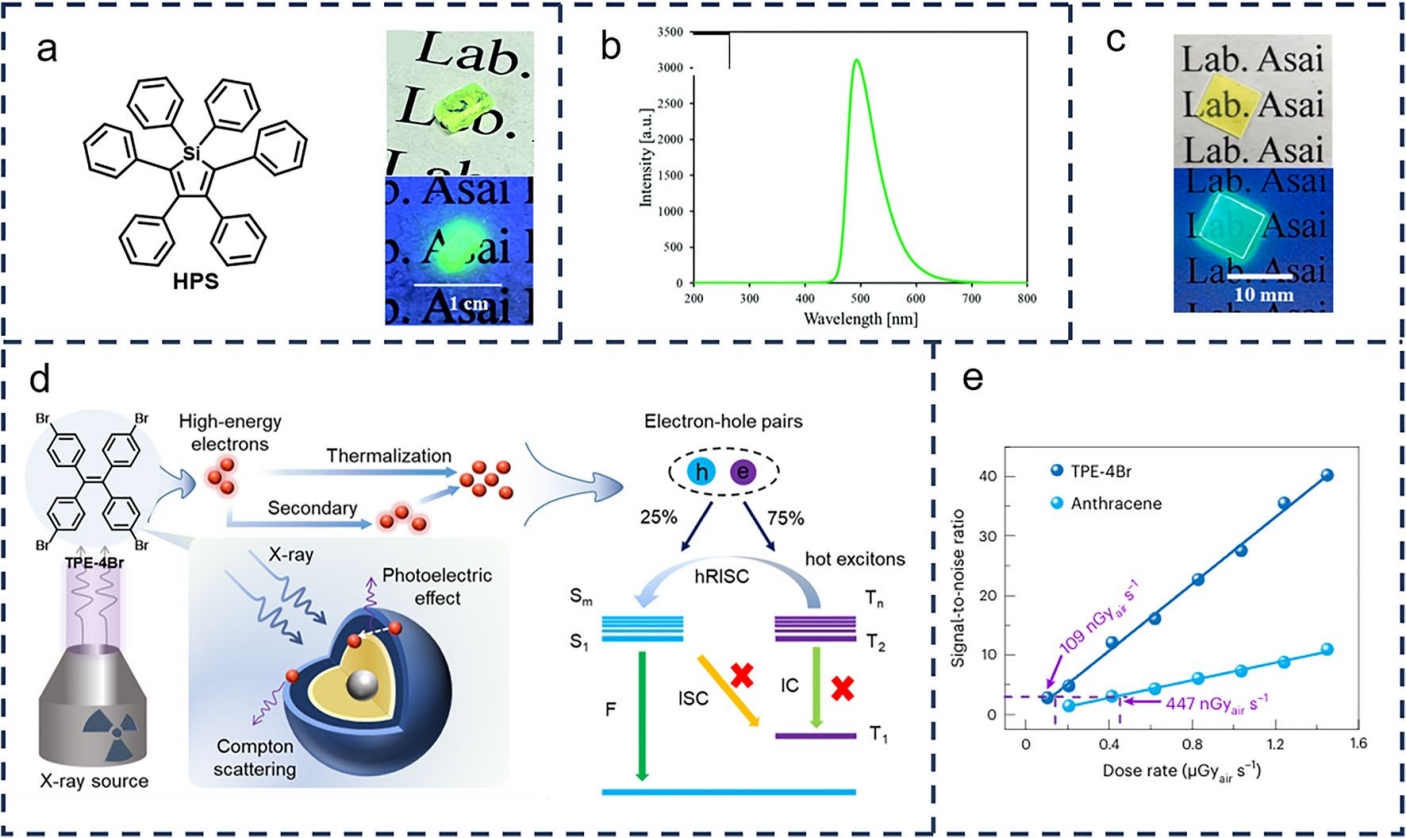
**a** Physical images of the molecular structure and crystals of HPS under natural light and UV light; **b** XRL spectra of HPS crystals. (**a** and **b** Reproduced with permission from Ref. [37] Copyright 2022 the Partner Organizations). **c** Photographs of synthetic plastic scintillators prepared using HPS under visible light (top) and UV (*λ* = 302 nm) irradiation (bottom). (**c** Reproduced with permission from Ref. [38] Copyright 2023 Elsevier B.V.). **d** Schematic representation of the X-ray induced scintillation process in a TPE-4Br thermal exciton scintillator. h, hole; e, electron; F, fluorescence; ISC, intersystem crossing. **e** Signal-to-noise ratio as a function of the dose rate for TPE-4Br and anthracene. (**d** and **e** Reproduced with permission from Ref. [39] Copyright 2024 Nature)

One notable advancement is in hydroxyphenyl silane (HPS) crystals, which emit a strong peak at 490 nm under X-ray excitation, with a scintillation decay time of 21 ns and a light yield of 11,000 photons MeV^−1^ (Fig. 3a, b) [37]. Researchers have further optimized these materials by doping HPS into poly(vinylcarbazole) (PVK), creating a series of plastic scintillators with varying concentrations of HPS (as shown in Fig. 3c). At HPS concentrations of 0.50 and 1.0 mol%, these scintillators achieve a scintillation light yield of 8200 photons MeV^−1^, demonstrating the tunability of AIE materials for improved performance in X-ray detection [38].

The effectiveness of X-ray detection is heavily influenced by the atomic composition of the materials used. Materials with higher atomic numbers typically exhibit superior X-ray absorption and attenuation efficiencies. One approach to further enhance the X-ray absorption capability and luminescence intensity of AIE materials is the introduction of heavy atoms, such as halogen doping, which can significantly improve detection sensitivity. For example, Du et al. [39] developed a novel strategy to create highly efficient and ultra-fast organic scintillators. By directing all thermal excitons into a rapid singlet emission state—bypassing the lowest triplet state—they synthesized 1,1,2,2-tetraphenylethylene(4-bromophenyl) (TPE-4Br) (Fig. 3d). This scintillator exhibits remarkable properties, including an ultra-fast radiative lifetime, high light yield, and enhanced X-ray absorption efficiency. Notably, TPE-4Br demonstrated a minimum detection limit of 109 nGy s^−1^, which is 50 times lower than the conventional dose rate required for medical diagnoses (5.5 μGy s^−1^), highlighting its potential for highly sensitive X-ray detection (Fig. 3e).

**Fig. 4:**
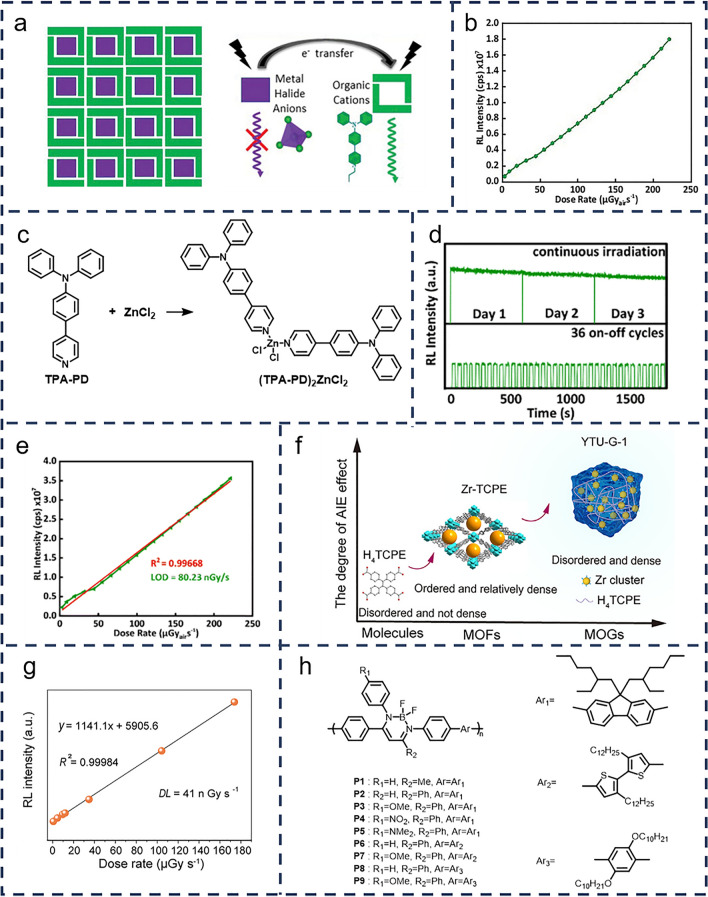
**a** Design of a 0D organic metal halide hybrid containing metal halide polyhedrons (ZnBr_4_^2−^, purple squares) fully isolated and surrounded by AIE organic cations (TPA-P^+^, green rectangles), and mechanism of sensitized radioluminescence. **b** Dependence of radioluminescence on the radiation dose rate for (TPA-P)_2_ZnBr_4_. (**a** and **b** Reproduced with permission from Ref. [40] Copyright 2023 Wiley–VCH GmbH). **c** AIE organic molecule 4-(4-(diphenylamino)phenyl)-1-(propyl)pyridinium (TPA-PD) reacts with zinc chloride (ZnCl_2_) to form the organometallic metal halide complex (TPA-PD)_2_ZnCl_2_. **d** Radioluminescence stability at 448 nm for (TPA-PD)_2_ZnCl_2_ under continuous irradiation (top) and repeated on–off cycles of X-rays (bottom) at a dose rate of 221.39 μGy s^−1^; **e** Dose–response linearity measurement of (TPA-PD)_2_ZnCl_2_. (**d** and **e** Reproduced with permission from Ref. [41] Copyright 2024 The Royal Society of Chemistry). **f** Construction of highly luminescent metal organogel (MOG), YTU-G-1; **g** Normalized radioluminescence intensity with different X-ray dose rates for YTU-G-1 in which the slope represents the sensitivity for X-ray. (**f** and **g** Reproduced with permission from Ref. [42] Copyright 2024 Elsevier Inc). **h** A series of boron diimide copolymers with different substituents and comonomers synthesized by Suzuki–Miyaura coupling

In a different approach, Shonde et al. [40] further advanced the field by combining high-Z metal halide anions with highly luminescent AIE organic cations to create robust organic–inorganic hybrid systems. These hybrid systems, unlike the halogen-doped systems discussed earlier, exhibit both enhanced X-ray absorption and rapid sensitized radioluminescence. They synthesized the one-dimensional organic metal halide (TPA-P)_2_ZnBr_4_ (Fig. 4a), which demonstrated an absolute light yield of (14,700 ± 800) photons MeV^−1^ and a decay lifetime of 9.96 ns. As illustrated in Fig. 4b, this organic metal halide hybrid scintillator also showed a low detection limit of 21.3 nGy s^−1^ and excellent response linearity over a broad range of X-ray dose rates. Building on this, the team developed an efficient molecular scintillator based on the AIE organic zinc halide complex (TPA-PD)_2_ZnCl_2_ (Fig. 4c) [41]. This covalent organic metal halide complex not only exhibited effective X-ray absorption due to the presence of high-Z metal halides but also exhibited rapid and efficient radioluminescence in the solid state thanks to its AIE characteristics. As demonstrated in Fig. 4d, e, (TPA-PD)_2_ZnCl_2_ exhibited good radiation stability, remaining stable after 36 on–off cycles, and achieved a remarkably low detection limit of 80.23 nGy s^−1^, making it one of the most efficient molecular scintillators developed to date.

In another significant study, Liu’s team developed a densely structured and rigid metal–organic gel scintillator, YTU-G-1 (Fig. 4f), incorporating the AIE-active molecule H_4_TCPE [42]. As shown in Fig. 4g, this innovative scintillator demonstrates a high quantum efficiency of 95.5% and an exceptionally low radiation detection limit of 41 nGy s^−1^, highlighting the continued advancements of AIE materials in the field of radiation detection.

Importantly, the application of AIE materials extends beyond small molecules and metal-doped complexes. For instance, Tanaka et al. [43] developed a film-type scintillator based on conjugated polymers with AIE properties (Fig. 4h). The team synthesized a series of boron diimide copolymers, varying the substituents and comonomers to optimize performance. These polymers not only exhibited characteristic AIE behavior but also emitted distinct luminescence colors under X-ray excitation, a crucial feature for detecting radiation across different energy levels. This advancement underscores the versatility of AIE materials in X-ray detection and imaging.

Table 1 summarized the radiation detection properties of representative aggregation-induced emissive scintillators. Compared with the traditional organic and inorganic scintillators, AIE materials possess unique properties, including efficient X-ray absorption, high light yield, and low detection limits [44–53]. For instance, while inorganic scintillators such as SrI_2_:Eu exhibit a high light yield of 93,000 photons/MeV, they suffer from relatively long decay times (1200 ns), limiting their performance in fast response applications [49]. In contrast, AIE scintillators like TPE-4Br, despite their lower light yield of 34,600 photons MeV^−1^, demonstrate a significantly faster decay time of 1.79 ns and a high PLQY of 93.7%. This combination of rapid response and high quantum yield ensures superior detection efficiency. In addition, AIE materials also offer immense potential for performance optimization through tunable molecular structures, making them potential candidates for next-generation radiation detection technologies.

**Table 1:** Some systems containing AIE molecules have excellent radiation detection properties.

AIE molecule included	Light yield (photons MeV^−1^)	τ_PL_(ns)	PLQY (%)	Limit of detection (nGy s^−1^)	References
	11,000	6.2	^1^72.3	–	[37, 38]
	34,600	1.79	93.7	109	[39]
	14,700 ± 800	9.96	71	21.3	[40]
	13,423	1.81	65	80.23	[41]
	–	4.10	95.5	41	[42]
	–	^2^0.57	^2^11	–	[43]
	–	1.73	95.5	44	[53]
	–	7.2×10^3^	90	34.6	[54]

^1^10 mol% HPS-doped PVK

^2^Data from polymer P1

### Radiation Imaging

X-ray imaging is a technique that leverages the ability of X-rays to penetrate objects and their subsequent detection to generate detailed images of the internal structures of those objects [54–58]. This technology is widely used in various fields, including medical diagnostics, industrial inspection, security screening, and scientific research [59–61]. AIE materials, which exhibit enhanced luminescence in their aggregated state, offer advantages such as simple synthesis, low cost, and high fluorescence quantum yield. These properties significantly improve imaging quality by increasing signal intensity, while their short fluorescence lifetime enhances temporal resolution and the signal-to-noise ratio of imaging systems. As a result, AIE materials hold great promise for advancing radiation imaging technologies.

**Fig. 5:**
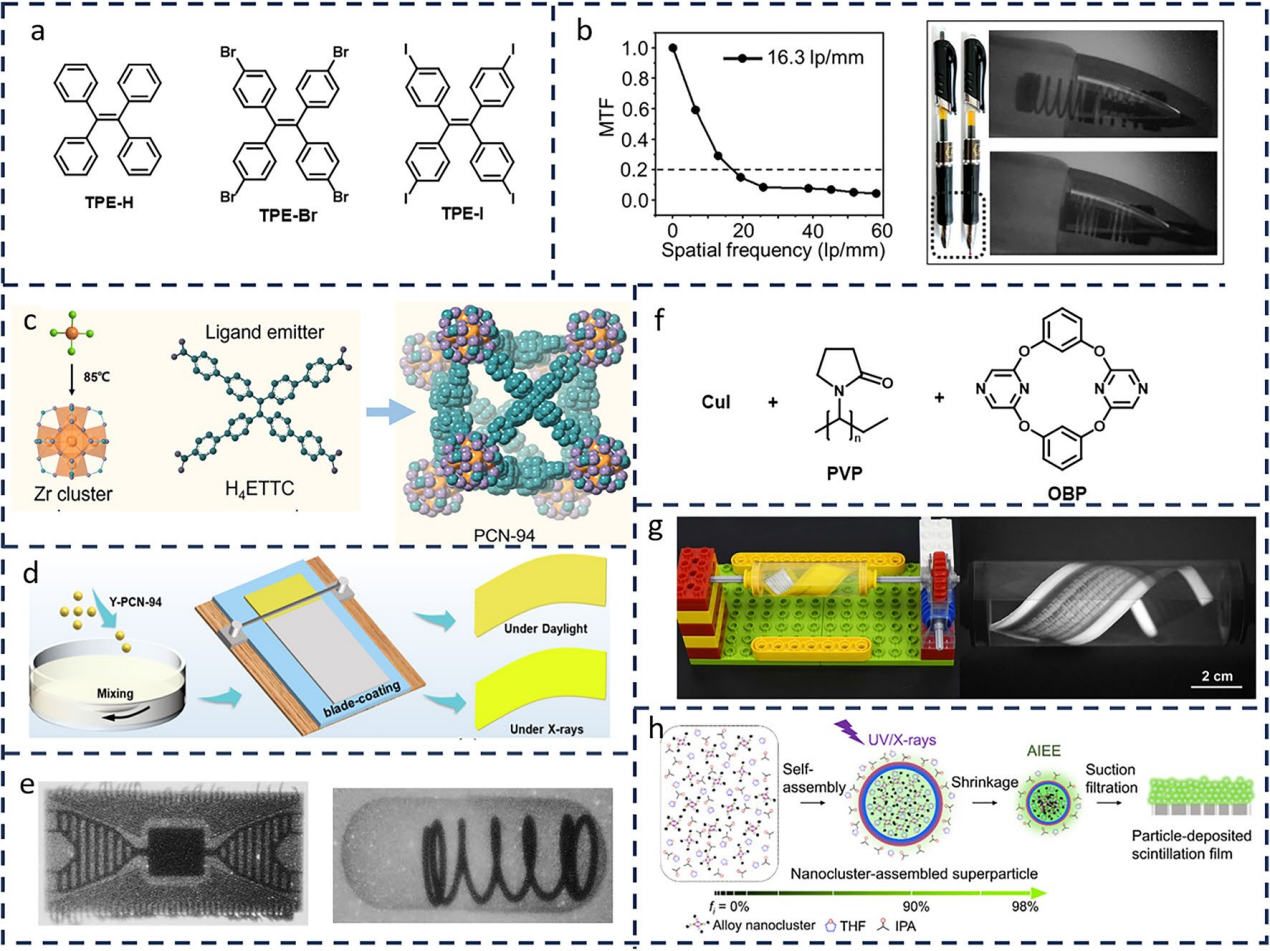
**a** A range of AIE materials containing halogen atoms: TPE-H, TPE-Br, TPE-I. **b** Modulation transfer function (MTF) of TPE-Br film X-ray image (PSF: 10 wt% of the composite) (left), bright and dark field photos of the pen (right). (**b** Reproduced with permission from Ref. [62] Copyright 2022 American Chemical Society). **c** PCN-94 crystal structure formed by Zr clusters and the organic ligand H_4_ETTC. **d** Schematic representation of the preparation of the composite Y-PCN-94@PDMS membrane. **e** X-ray imaging of a chipboard (left) and a spring-loaded pill (right) by using the composite Y-PCN-94@PDMS scintillating membrane. (**c–e** Reproduced with permission from Ref. [63] Copyright 2023 American Chemical Society). **f** Molecular structures contained in metal–organic framework (MOF) scintillators based on copper iodide clusters. **g** Dynamic flexible X-ray imaging device and real-time imaging photographs of flexible OLED display. (**g** Reproduced with permission from Ref. [64] Copyright 2023 Wiley–VCH GmbH). **h** Novel 0D scintillator Cu_2_Au_2_(R-BTT)_4_ based on phosphorescent AIEE-active metal nanoclusters and its particle deposition scintillation film preparation process. (**h** Reproduced with permission from Ref. [65] Copyright 2023 American Chemical Society)

Due to the challenges associated with the practical use of powder samples in X-ray imaging, researchers have turned to polysulfone (PSF), a highly flexible material, as the substrate. They then doped a series of halogen-containing AIE materials to create thin films suitable for X-ray imaging applications (Fig. 5a) [62]. As shown in Fig. 5b (left), the resulting film achieved an impressive X-ray imaging resolution of 16.3 lp mm^−1^, making it a promising candidate for X-ray photography and security screening. Furthermore, the TPE-Br scintillator screen demonstrated clear visibility of the internal structure of steel springs inside a pen (Fig. 5b, right).

The lack of high atomic number components limits the ability of some materials to effectively block X-rays, resulting in weak X-ray absorption and limited light output. To address these limitations, new scintillator materials or strategies must be developed. One promising direction is the design of organic–inorganic hybrid multi-component scintillators. For instance, Zhang et al. [63] reported a flexible composite scintillating film with excellent imaging performance. This film was created by embedding AIE luminophore (H_4_ETTC)-functionalized MOF scintillators (Y-PCN-94) into a polymer matrix (PDMS) (Fig. 5c). Notably, Y-PCN-94 demonstrates a strong AIE effect under both UV and X-ray irradiation, marking the first observation of AIE behavior in a MOF system under ionizing radiation. Combining the AIE effect with strong X-ray blocking ability, this material exhibits outstanding radioluminescence characteristics, such as a low X-ray detection limit (1.6 μGy s^−1^) and high imaging resolution (> 14.3 lp mm^−1^). The authors also fabricated a flexible composite X-ray scintillating film using PDMS and Y-PCN-94 through a simple blade-coating technique. In this Y-PCN-94@PDMS composite film, the internal components of wood chips and spring balls can be clearly displayed (Fig. 5d, e). Similarly, Peng et al. [64] developed an AIE-characteristic cyclic bridge ligand iodinated copper cluster MOF scintillator (Fig. 5f) with high X-ray excited luminescence (XEL) efficiency and excellent chemical stability. By adding PVP to prepare regular rod-shaped microcrystals, both XEL and processability were enhanced. This microcrystal was then used to fabricate a flexible scintillator screen capable of high-performance X-ray imaging in humid environments, achieving dynamic flexible X-ray imaging for the first time, with a resolution as high as 20 lp mm^−1^. This system enables real-time monitoring of the internal structure of flexible objects (Fig. 5g). Huang et al. [65] also explored a new class of 0D scintillators based on phosphorescent AIEE-active metal nanoclusters, Cu_2_Au_2_(R-BTT)_4_, and prepared particle-deposited scintillating films of varying thicknesses, achieving a spatial resolution as high as 28.4 lp mm^−1^.

## Applications of AIE Scintillators in Other Radiation Detection

### γ-Ray Detection

γ rays, the electromagnetic waves with the shortest wavelengths, possess the highest photon energy, ranging from thousands of electron volts to approximately 8 million electron volts, and exhibit the strongest penetrating ability. Due to these properties, γ-rays are widely used in applications such as interplanetary exploration, non-contact industrial sensors, and cancer therapy [66, 67]. When γ-rays pass through a detector, they may be absorbed, scattered, or interact with the detector material through Compton scattering. This interaction produces signals like scintillation light and charge carriers, which are then converted into electrical signals and recorded [68]. Traditional *γ*-ray detectors, typically based on scintillating crystals or high-performance semiconductor materials [69–71], such as NaI(Tl) and CdZnTe, offer high detection efficiency. However, these materials often come with complex fabrication processes, high costs, and limited flexibility.

**Fig. 6:**
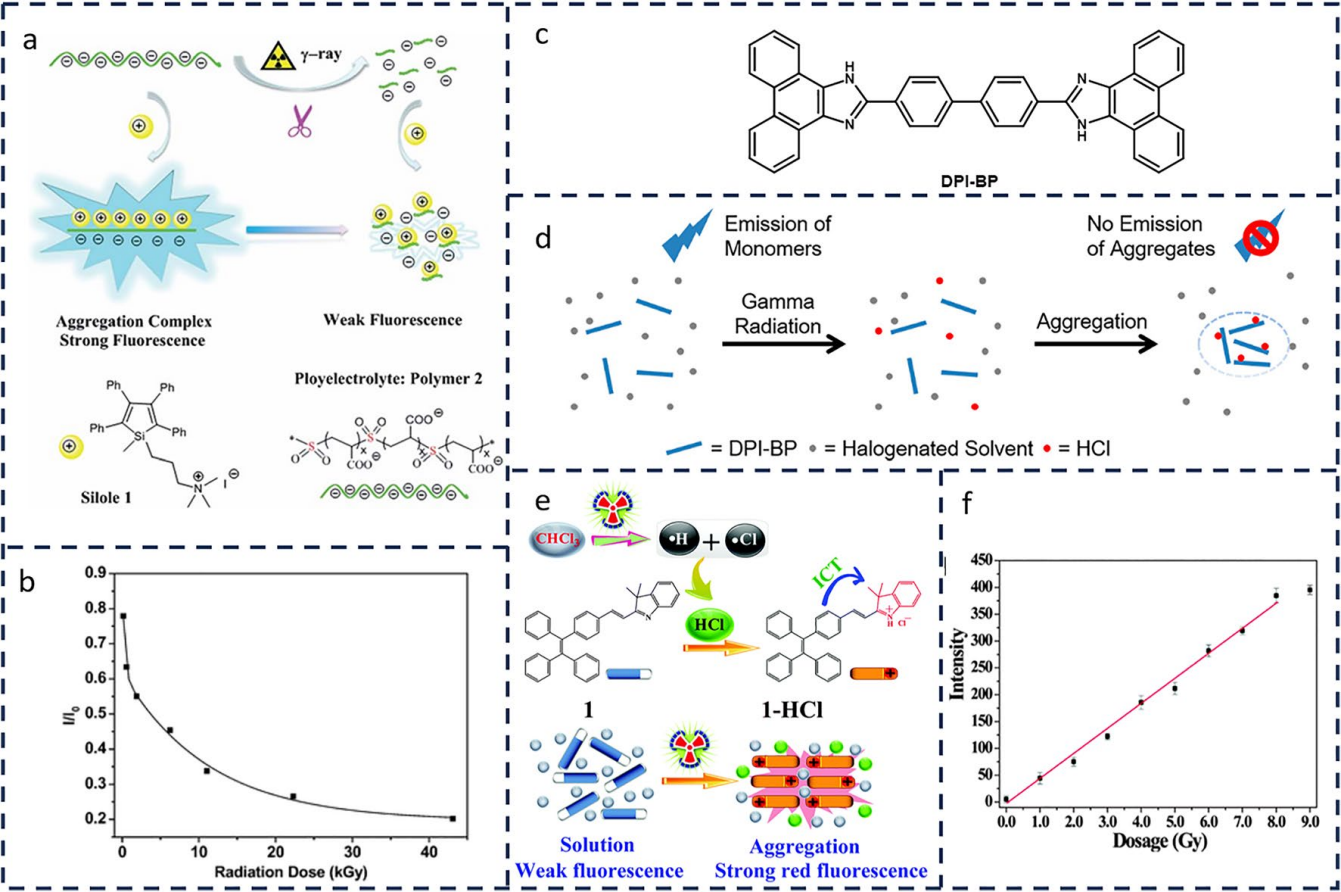
**a** γ-ray fluorescence detection design based on silicene 1 and polymer 2. **b** Variation of fluorescence intensity at 465 nm of the aqueous solution of silole 1 (1.0 × 10^–5^ M) and polymer 2 (15 mg L^−1^) with increasing γ-ray radiation dose. (**a** and **b** Reproduced with permission from Ref. [72] Copyright 2011 The Royal Society of Chemistry). **c** Molecular structure of DPI-BP. **d** Schematic drawing of the fluorescence sensing mechanism of DPI-BP. Protonation interaction with HCl generated by radiation causes molecular aggregation of DPI-BP, leading to fluorescence quenching due to π–π stacking. (**d** Reproduced with permission from Ref. [78] Copyright 2014 American Chemical Society). **e** Chemical structures of compound 1 and 1·HCl and design rationale for the fluorescence turn-on detection of γ-radiation. **f** A plot showing the fluorescence intensity at 640 nm *vs.* the dosage of γ-rays for the CHCl_3_ solution of 1 (3.0 mL, 10.0 μM); the data in the range of 0.0–8.0 Gy were fitted using the following equation: *Y* = 46.37351*X* − 6.48515, *R*^2^ = 0.991. (**e** and **f** Reproduced with permission from Ref. [79] Copyright 2015 The Royal Society of Chemistry)

In response to these limitations, AIE materials, recognized for their superior optical properties, have emerged as promising candidates for novel *γ*-ray detection materials. Liu et al. [72] successfully developed a simple fluorescent detection technique based on the principle of aggregation-induced emission (AIE) for γ-ray detection. As illustrated in Fig. 6a, this technique exploits the AIE characteristics of siloxane compound 1 and the vulnerability of the sulfonyl groups (-SO_2_-) in the main chain of polymer 2 to degradation under γ-ray irradiation, enabling effective γ-ray detection. Furthermore, this detection method operates under ambient conditions and can sensitively detect γ-ray doses as low as 0.13 kGy (Fig. 6b). Under γ radiation, halogenated solvents such as chloroform (CHCl_3_) and dichloromethane (CH_2_Cl_2_) decompose into free radicals [73, 74]. These radicals are highly reactive and quickly recombine to form new compounds, including hydrochloric acid (HCl). Notably, water (humidity) and oxygen in ambient conditions have little effect on HCl production because their reactions with free radicals are slow [75–77].

Building on this principle, Zang et al. [78] developed a novel fluorescent sensor molecule, DPI-BP (Fig. 6c), with a sensing mechanism primarily based on the addition reaction between HCl and the imidazole groups of DPI-BP. As shown in Fig. 6d, the HCl adduct of DPI-BP undergoes aggregation, leading to the quenching of its molecular fluorescence due to intermolecular π-π stacking. This aggregation allows the sensor to rapidly detect the HCl produced under γ radiation. The sensor significantly enhances the detection limit of γ radiation by three orders of magnitude, reducing it to 0.01 Gy. Building on this, Dong et al. [79] harnessed the AIE characteristics of the TPE unit in combination with the chemical behavior of CHCl_3_, which decomposes to generate HCl under γ-ray irradiation. This protonates compound 1 and promotes its aggregation, yielding a γ radiation detection limit of 0.023 Gy, thus enabling highly sensitive γ-ray detection (Fig. 6e, f).

The key advantage of AIE materials in γ-ray detection lies in their ability to enhance the detection response through molecular design. Additionally, the low cost and solution processability of AIE materials make them attractive candidates for large-scale γ-ray detectors, especially for portable and flexible designs—an important factor for practical applications.

### Fast Neutron Detection

The principle behind fast neutron detection relies on the interaction between high-energy fast neutrons and hydrogen nuclei in the scintillator material. After passing through the target object, fast neutrons undergo elastic scattering with hydrogen nuclei, resulting in recoil protons. These recoil protons deposit energy in the scintillator, which in turn generates electron–hole pairs, leading to the excitation of the scintillator and the emission of visible fluorescence. This process effectively converts the neutron field distribution into a visible light pattern, which is subsequently captured and recorded by the optical system, enabling fast neutron detection and imaging [80]. Due to their strong penetrating power, fast neutrons can traverse most materials, making fast neutron detection a promising technique for non-destructive testing of large components. This technology holds significant potential in various fields, including aerospace, nuclear energy, and advanced manufacturing [81–84].

**Fig. 7:**
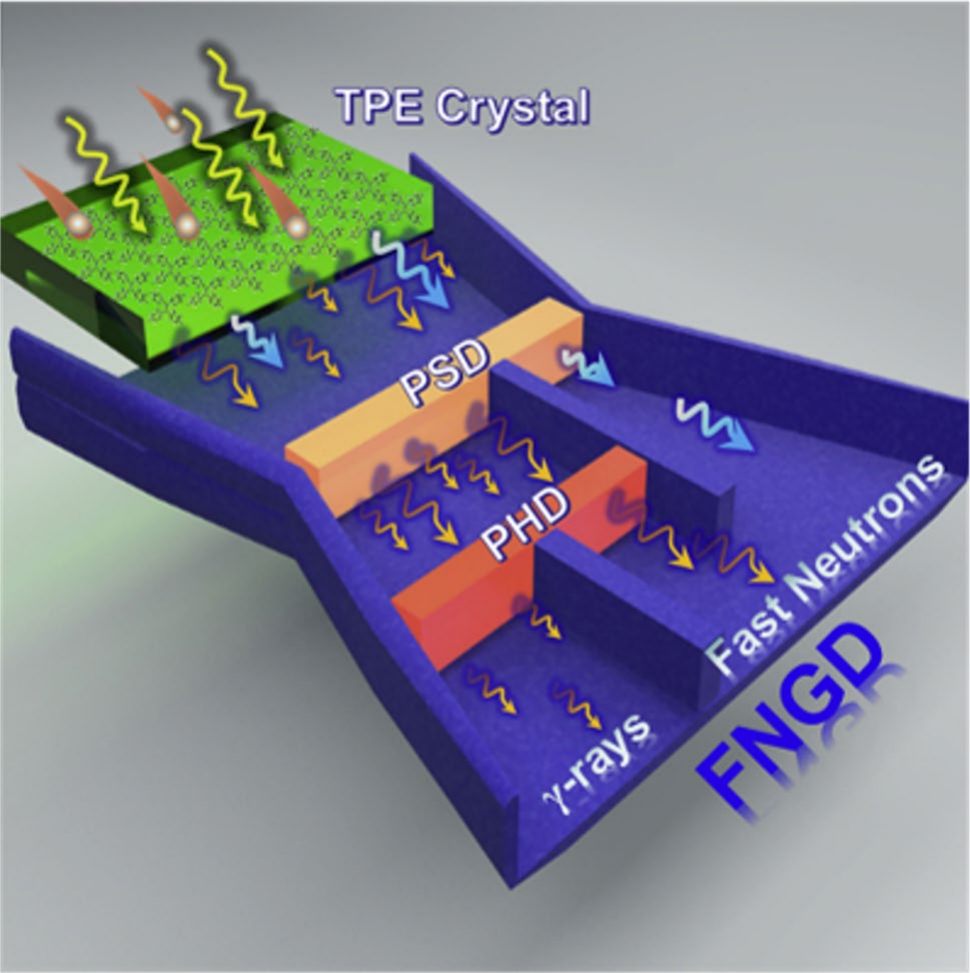
Schematic of fast neutron/γ discrimination with TPE single crystal. (Reproduced with permission from Ref. [85] Copyright 2022 Elsevier Inc.)

In this context, Sun et al. [85] introduced a “dual discrimination” concept using TPE-based single-crystal scintillators (Fig. 7) to effectively distinguish fast neutrons from the accompanying γ noise. By employing pulse shape discrimination (PSD) and pulse height discrimination (PHD), they achieved a high level of separation between fast neutrons and γ rays, thereby enhancing the fast neutron count rate.

The application of AIE materials in fast neutron detection is still in its early stages but shows considerable promise. A key challenge lies in designing AIE molecules optimized for neutron detection, particularly with high hydrogen content, as hydrogen atoms efficiently interact with fast neutrons. Neutron detection also demands materials with high radiation stability, as exposure to high-energy particles like fast neutrons can cause significant damage.

To address these challenges, integrating AIE materials with neutron-absorbing components, such as boron or lithium, or designing hydrogen-rich AIE molecules can enhance their interaction with fast neutrons. This interaction generates secondary particles, such as alpha particles or protons, which excite the AIE materials to emit detectable fluorescence signals. The chemical tunability of AIE materials further enables molecular engineering to optimize their neutron interaction efficiency and optical response, improving overall detector performance. Compared to traditional detectors, AIE-based systems offer the potential for superior stability, higher sensitivity, and adaptability to complex detection environments, positioning them as promising candidates for next-generation neutron detection technologies.

## Conclusions and Prospects

The unique properties of AIE materials have led to revolutionary advancements in the field of radiation detection and imaging. Unlike traditional luminescent materials, AIE materials exhibit enhanced luminescence efficiency in their aggregated state, offering significant advantages for various applications, including X-ray, γ-ray, fast neutron detection, and radiation imaging. Furthermore, the chemical diversity of AIE materials provides vast opportunities for designing novel detectors. By utilizing strategies such as molecular structure optimization, metal doping, and polymer incorporation, it is possible to develop detectors with superior AIE-based radiation detection capabilities. This advances radiation detection technology toward higher sensitivity, resolution, and stability.

However, despite these promising advancements, several challenges need to be addressed. The stability of AIE materials under prolonged exposure to high-energy radiation, such as X-rays and γ-rays, remains a critical concern. Accumulation of radiation-induced defects can quench luminescence and degrade performance, particularly in comparison to the inherent robustness of traditional inorganic scintillators. Additionally, while AIE materials exhibit high photoluminescence quantum yields and short lifetimes, their light yield has yet to surpass that of conventional organic and inorganic scintillators, limiting their competitiveness in applications requiring high sensitivity and resolution. Challenges in fabrication, including poor film formation, non-uniformity, and scalability, also hinder their development for large-area detectors. Furthermore, enhancing detection capabilities for challenging radiation types, such as fast neutrons and α particles, remains an area requiring further exploration.

Looking ahead, the future of AIE materials in radiation detection lies in advancing molecular design strategies and exploring novel composite systems. Strategies such as optimizing molecular structures, incorporating metal doping, and integrating AIE-active molecules with stable inorganic components can improve radiation tolerance, mitigate luminescence degradation, and enhance overall performance. Additionally, developing scalable and cost-effective fabrication methods, such as inkjet printing or spray-coating, will facilitate the production of large-area detectors with consistent properties. Exploring multifunctional materials and hybrid systems will further expand detection capabilities, offering solutions for diverse and complex radiation environments. Continued progress in material science and detection technologies will position AIE materials as pivotal components in next-generation radiation detection systems, addressing critical needs in health monitoring, security screening, and scientific research.
